# Have Niche, Will Travel. New Means of Linking Diet and Ecomorphology Reveals Niche Conservatism in Freshwater Cottoid Fishes

**DOI:** 10.1093/iob/obz023

**Published:** 2019-09-06

**Authors:** T J Buser, D L Finnegan, A P Summers, M A Kolmann

**Affiliations:** 1 Department of Fisheries and Wildlife, Oregon State University, Corvallis, OR 97321, USA; 2 Department of Biology, Western Washington University, Bellingham, WA 98225, USA; 3 Department of Biology and SAFS, University of Washington s Friday Harbor Laboratories, Friday Harbor, WA 98250, USA; 4 Department of Biological Sciences, George Washington University, Washington, DC 20052, USA

## Abstract

Evolutionary transitions between habitats have been catalysts for some of the most stunning examples of adaptive diversification, with novel niches and new resources providing ecological opportunity for such radiations. In aquatic animals, transitions from saltwater to freshwater habitats are rare, but occur often enough that in the Neotropics for example, marine-derived fishes contribute noticeably to regional ichthyofaunal diversity. Here, we investigate how morphology has evolved in a group of temperate fishes that contain a marine to freshwater transition: the sculpins (Percomorpha; Cottoidea). We devised a novel method for classifying dietary niche and relating functional aspects of prey to their predators. Coupled with functional measurements of the jaw apparatus in cottoids, we explored whether freshwater sculpins have fundamentally changed their niche after invading freshwater (niche lability) or if they retain a niche similar to their marine cousins (niche conservatism). Freshwater sculpins exhibit both phylogeographical and ecological signals of phylogenetic niche conservatism, meaning that regardless of habitat, sculpins fill similar niche roles in either saltwater or freshwater. Rather than competition guiding niche conservatism in freshwater cottoids, we argue that strong intrinsic constraints on morphological and ecological evolution are at play, contra to other studies of diversification in marine-derived freshwater fishes. However, several intertidal and subtidal sculpins as well as several pelagic freshwater species from Lake Baikal show remarkable departures from the typical sculpin bauplan. Our method of prey categorization provides an explicit, quantitative means of classifying dietary niche for macroevolutionary studies, rather than relying on somewhat arbitrary means used in previous literature.

## Synopsis

Tem Nicho, Viaja. Novos Meios de Associar Dieta e Ecomorfologia Revelam Conservadorismo de Nicho em Peixes Cotoides de Água Doce (Have Niche, Will Travel. New Means of Linking Diet and Ecomorphology Reveals Niche Conservatism in Freshwater Cottoid Fishes) Transições evolutivas entre habitats têm sido catalisadores de alguns dos mais impressionantes exemplos de diversificação adaptativa, com novos nichos e recursos proporcionando oportunidade ecológica para tais radiações. Em animais aquáticos, as transições de água salgada para habitats de água doce são raras, mas ocorrem com freqüência suficiente para que, nos Neotrópicos, por exemplo, os peixes marinhos contribuam notavelmente para a diversidade regional da ictiofauna. Aqui, nós investigamos como a morfologia evoluiu em um grupo de peixes temperados que contêm uma transição marinha para a água doce: os esculpentes (Percomorpha; Cottoidea). Nós concebemos um novo método para classificar o nicho alimentar e relacionar os aspectos funcionais das presas aos seus predadores. Juntamente com medidas funcionais do aparato de mandíbula em cotoides, exploramos se os esculpentes de água doce mudaram fundamentalmente seu nicho depois de invadi-la (labilidade de nicho) ou se eles mantêm um nicho semelhante aos seus primos marinhos (conservadorismo de nicho). Os esculpentes de água doce exibem sinais filogeográficos e ecológicos de conservadorismo filogenético de nicho, o que significa que, independente do habitat, os esculpentes preenchem papéis ecológicos semelhantes em água salgada ou doce. Mais do que a concorrência guiando o conservadorismo de nicho em cotoides de água doce, argumentamos que fortes restrições intrínsecas à evolução morfológica e ecológica estão em jogo, em contraste com outros estudos de diversificação em peixes de água doce derivados do mar. No entanto, vários esculpentes intertidais e subtidais, bem como várias espécies pelágicas de água doce do Lago Baikal, mostram notáveis desvios do típico bauplan dos esculpentes. Nosso método de categorização de presas fornece um modo explícito e quantitativo de classificar o nicho alimentar para estudos macroevolutivos ao invéz de depender de meios arbitrários usados na literatura anterior. Translated to Portuguese by G. Sobral (gabisobral@gmail.com)

## Introduction

Invasion of a novel environment precedes many of the most lamented and lauded animal success stories. The explosive population growth of some anthropogenic invasive species has had dire consequences for their new habitats (e.g., zebra mussels, cane toads), and their success illustrates the opportunity that new habitats pose for species able to exploit them. On evolutionary timescales, transitions between habitats have heralded prodigious diversification in some taxa (e.g., Hawaiian silverswords, *Tetragnatha* spiders; Robichaux et al. 1990; Gillespie 2004) and in the familiar cases of Galapagos finches and African rift lake cichlids, diversification of diet is closely followed by adaptation of morphological characters involved with capture and processing of prey (Schluter and Grant 1984; Grant and Grant 1989; Takahashi et al. 2007; Cooper et al. 2010). This exploration and accompanying specialization on prey resources contained in novel habitats can produce a radiation of morphotypes adapted to fit those opportunities (Gavrilets and Losos 2009; Slater and Friscia 2019).

Freshwater habitats present this kind of ecological opportunity for marine lineages that are able to invade them. After transitioning to freshwater, an invading lineage either retains their ancestral morphology and ecology (phylogenetic niche conservatism; Wiens and Donoghue 2004; Wiens and Graham 2005) or radiates to take advantage of novel resources (phylogenetic niche lability; Wiens et al. 2006). Exploration of novel niches depends on whether diversification is curtailed by competition with entrenched indigenous taxa (Betancur-R et al. 2012; Bloom and Lovejoy 2012) or by intrinsic constraints on the invaders themselves, i.e., their adaptability (Lee et al. 2007). Freshwater invasions occur in tropical, temperate, and boreal zones, but different patterns of abundance and frequency of invaders appear with latitude. For example, marine-derived lineages from higher latitudes (e.g., salmonids, clupeids, galaxiids, cottids, osmerids) are dominant numerically and by biomass, despite contemporaneous or even more recent invasion of freshwater compared with their tropical counterparts (Yokoyama and Goto 2005; Ilves and Taylor 2008; Lecaudey et al. 2018).

One temperate and boreal lineage, the sculpins (superfamily Cottoidea), includes some 380 species of fishes (Smith and Busby 2014). While most sculpins are found in marine habitats, there is a large freshwater contingent (∼100 species), in which all but one evolved from a single ancestral invasion of freshwater. This large clade is distributed across the northern hemisphere and includes the radiation of sculpins endemic to Lake Baikal, Siberia. Baikal cottoids, which are nested in the genus *Cottus* but are nominally in other genera, have exploited a variety of seemingly novel niches, perhaps most remarkable among them being the pelagic, planktivorous Baikal oilfishes (*Comephorus* spp.). Another invader of freshwater is *Myoxocephalus thompsonii*, the deepwater sculpin, which shares a very recent (mid-Pleistocene) common ancestor with the marine, but highly freshwater tolerant species, *Myoxocephalus quadricornis* in the Nearctic (Kontula and Väinölä 2003; Sheldon et al. 2008).

The success and apparent adaptability of freshwater sculpins introduces a tantalizing question regarding their marine relatives: are freshwater sculpins exploiting new niches with novel morphologies; which is to say, are they exploiting freshwater and marine niches in fundamentally different ways? Alternatively, could there be a single sculpin bauplan that works well in both marine and freshwater environments? While it might be easy enough to compare the functional morphology of freshwater and marine species, e.g., *Cottus gobio* vs. *Oligocottus maculosus*, it is less clear how to compare the ecology of these species directly, given the drastic differences in geography and community structure of their respective habitats.

Typical diet classifications often include descriptive terms such as “insectivore,” “invertivore,” and “molluscivore.” While such descriptions may be appropriate in a geographically restricted study, those terms encompass very different sets of potential prey items in freshwater vs. marine environments, and this limits their usefulness in comparative studies (see Norton 1995). However, over-partitioning of prey items makes meaningful comparisons of diet across varied habitats difficult. For example, the pelagic amphipods of Lake Baikal (e.g., *Macrohectopus*) show remarkable morphological and ecological convergence with the pelagic mysids found in many marine systems (Takhteev 2000). It is conceivable then that these two groups would present similar challenges to would-be predators in their respective environments. A finer-scale categorization of diet (i.e., based on taxonomy of the prey items) would ignore the similarities of these distantly related taxa. This presents a kind of Goldilocks paradox of where to draw the line when delineating diet categories: categories that are too broad become meaningless when applied across disparate environments, but categories that are too narrow ignore functional commonalities in predator–prey interactions that may recur due to convergence. The qualitative nature of most categorizations exacerbates these issues further, in addition to making replication across studies difficult. Alternatively, a categorization of diet based on the morphology and behavior of potential prey items would lend itself to comparisons across disparate habitats and enable a quantitative means of grouping potential prey items into generalizable categories.

The goal of this study was to determine whether there is a relationship between the functional morphology of the feeding apparatus and the dietary ecology in cottoid fishes. To investigate this question, we first (1) inferred the phylogenetic relationships of cottoid taxa using previously published molecular sequence data. We (2) measured ecomorphological traits of each species from micro-computed tomographic scans, and (3) inferred dietary guilds using functional attributes of all known prey taxa for the sculpin species in our study. Finally, we (4) used phylogenetic comparative methods to test for a relationship between diet and morphology across taxa and tested for differences in freshwater vs. saltwater-dwelling species. Specifically, we were interested in whether freshwater sculpins retain ancestral phenotypes and ecological guilds (niche conservatism) or overlap, expand, or occupy novel regions of feeding morphospace relative to marine sculpins (niche lability). These novel regions may include unique morphologies as well as new configurations of traits and highlight particular cottoid taxa which have evolved away from ancestral bauplans when accessing novel prey resources.

## Materials and methods

### Taxon sampling

Freshwater species make up approximately one fourth of the superfamily Cottoidea (∼100/390). We selected 24 freshwater species and 30 marine species (∼10% of total cottoid species) for this study (Table 1). We used previously published phylogenetic hypotheses of sculpin relationships to inform our taxon sampling (Kinziger et al. 2005; Yokoyama and Goto 2005; Knope 2013; Smith and Busby 2014; Buser and López 2015; Girard and Smith 2016), with the aim of representing maximum clade diversity. Our taxonomy of marine sculpins follows that of Smith and Busby (2014) and includes representatives of the sculpin families: Agonidae, Cottidae, Jordaniidae, Psychrolutidae, Rhamphocottidae, and Scorpaenichthyidae. Our taxonomy of freshwater sculpins follows that of Kinzinger et al. (2005) and includes representatives of four (out of five) named clades within the *Cottus* radiation: Baikalian, Cottopsis, Cottus, and Uranidae. The taxon *Hexagrammos decagrammus* (family Hexagrammidae) is included as an outgroup.

**Table 1:** Primary habitat, taxonomic family, and sources of molecular sequence data for taxa included in this study. See the “Materials and methods” section for appropriate habitat references. Taxonomic family follows (Smith and Busby 2014)

			Molecular locus				
Taxon	Family	Primary habitat	12s	16Sar-br	ATPase8and6	COI	Cytb
*Abyssocottus korotneffi*	Cottidae	Freshwater			AY116310^1^		AY116342^1^
*Comephorus dybowskii*	Cottidae	Freshwater			AY116324^1^		AY116356^1^
*Cottocomephorus grewingki*	Cottidae	Freshwater			AY116327^1^		AY116359^1^
*Cottus aleuticus*	Cottidae	Freshwater	AB188191^2^		AY833273^3^	EU523991^4^	AF549106^5^
*Cottus asper*	Cottidae	Freshwater	MF326939^6^	EF458399^7^	AY833275^3^	EU523994^4^	AF549105^5^
*Cottus asperrimus*	Cottidae	Freshwater			AY833276^3^		AY833331^3^
*Cottus baileyi*	Cottidae	Freshwater			AY833277^3^		AY833332^3^
*Cottus bairdii*	Cottidae	Freshwater	KM057993^8^	AY539018^9^	AY833280^3^	JN025025^10^	AF549162^5^
*Cottus beldingii*	Cottidae	Freshwater			AY833285^3^	JN025028^10^	AF549116^5^
*Cottus carolinae*	Cottidae	Freshwater	KM057994^8^	AY539019^9^	AY833290^3^	JN025050^10^	AF549110^5^
*Cottus cognatus*	Cottidae	Freshwater	AB188190^2^	KJ778622^11^	AY116333^1^	EU523999^4^	NA^12^*
*Cottus confusus*	Cottidae	Freshwater		KJ010739^13^	AY833294^3^	KF918868^14^	AY833343^3^
*Cottus extensus*	Cottidae	Freshwater			AY833295^3^		AY833344^3^
*Cottus gobio*	Cottidae	Freshwater	AB188189^2^	KJ128752^15^	AY116334^1^	HQ960935^16^	AY116366^1^
*Cottus gulosus*	Cottidae	Freshwater			AY833299^3^	JN025103^10^	KJ509432^17^
*Cottus hubbsi*	Cottidae	Freshwater			AY833301^3^	JN025104^10^	AY833350^3^
*Cottus klamathensis*	Cottidae	Freshwater			AY833305^3^	JN025112^10^	AY833352^3^
*Cottus leiopomus*	Cottidae	Freshwater			AY833308^3^	HQ971431^10^	AY833355^3^
*Cottus perplexus*	Cottidae	Freshwater			AY833313^3^	JN025117^10^	AF549108^5^
*Cottus pitensis*	Cottidae	Freshwater			AY833314^3^	JN025122^10^	AY833360
*Cottus poecilopus*	Cottidae	Freshwater	AB188185^2^	AY539020^9^	AY116336^1^	HQ960875^16^	AY116370^1^
*Cottus pollux*	Cottidae	Freshwater	AB188176^2^	LC097787^18^	AY116337^1^	LC097835^18^	AY116368^1^
*Cottus rhotheus*	Cottidae	Freshwater			AY833317^3^	HQ579026^10^	AF549114^1^
*Cottus ricei*	Cottidae	Freshwater			AY833318^3^	JN025135^10^	AY833363^3^
*Blepsias cirrhosus*	Agonidae	Marine	KM057948^8^	KJ010714^13^		KP827340^19^	EU836702^20^
*Hemilepidotus jordani*	Agonidae	Marine	KM057959^8^	AY539021^9^	AY833324^3^	KP827339^19^	AY833367^3^
*Hemilepidotus zapus*	Agonidae	Marine	KM057960^8^	AY539022^9^		HQ712450^21^	NA^12^*
*Hemitripterus bolini*	Agonidae	Marine	KM057962^8^	KM057862^8^		KP827342^19^	KM057904^8^
*Leptocottus armatus*	Cottidae	Marine	AB188194^2^	EF119251^7^	AY833323^3^	FJ164714^22^	AF549104^5^
*Hexagrammos decagrammus*	Hexagrammidae	Marine		AY539011^9^		FJ164640^22^	
*Jordania zonope*	Jordaniidae	Marine		AY539024^9^			NA^12^*
*Artedius fenestralis*	Psychrolutidae	Marine	KM057943^8^	AY539017^9^		JQ353989^23^	EU836698^20^
*Chitonotus pugetensis*	Psychrolutidae	Marine		EF119246^7^		KP827356^19^	EF521368^24^
*Clinocottus acuticeps*	Psychrolutidae	Marine				KP827297^19^	EF521387^24^
*Clinocottus analis*	Psychrolutidae	Marine	KM057950^8^	AY835646^25^	AY833272^3^	JN024969^10^	AY833327^3^
*Clinocottus embryum*	Psychrolutidae	Marine				KP827261^19^	EF521386^24^
*Clinocottus globiceps*	Psychrolutidae	Marine				KP827273^19^	EF521384^24^
*Clinocottus recalvus*	Psychrolutidae	Marine		AY583125^26^		KP827270^19^	EF521385^24^
*Dasycottus setiger*	Psychrolutidae	Marine	KM057955^8^	AY539040^9^		FJ164544^22^	
*Enophrys bison*	Psychrolutidae	Marine	KM057956^8^	EF119332^7^		GU440314^27^	EU836693^20^
*Gymnocanthus galeatus*	Psychrolutidae	Marine		KM057861^8^		HQ712423^21^	JQ406201^28^
*Icelinus filamentosus*	Psychrolutidae	Marine	KM057965^8^	AY539023^2^		FJ164691^22^	NA^12^*
*Icelus spiniger*	Psychrolutidae	Marine	KM057966^8^	KM057863^8^		HQ712508^21^	KM057905^8^
*Microcottus sellaris*	Psychrolutidae	Marine	KM057972^8^	AY539026^9^			KM057906^8^
*Myoxocephalus polyacanthocephalus*	Psychrolutidae	Marine	KM057974^8^	AY539027^9^	AY339242^29^	HQ712665^21^	AY338280^29^
*Oligocottus maculosus*	Psychrolutidae	Marine				KP827299^19^	EF521379^24^
*Oligocottus rimensis*	Psychrolutidae	Marine				KP827319^19^	EF521380^24^
*Oligocottus snyderi*	Psychrolutidae	Marine		KM057865^8^		KP827306^19^	EU836695^20^
*Orthonopias triacis*	Psychrolutidae	Marine	KM057977^8^	KM057867^8^			EF521370^24^
*Porocottus camtschaticus*	Psychrolutidae	Marine	KM057981^8^	KM057871^8^			KM057908^8^
*Psychrolutes phrictus*	Psychrolutidae	Marine	KM057982^8^	KM057872^8^		FJ165065^22^	KM057909^8^
*Triglops scepticus*	Psychrolutidae	Marine	KM057992^8^	AY539030^9^		KP827337^19^	NA^12^*
*Rhamphocottus richardsonii*	Rhamphocottidae	Marine	KM057985^8^	AY539015^9^		GU440501^27^	NA^12^*
*Scorpaenichthys marmoratus*	Scorpaenichthyidae	Marine	KM057987^8^	AY835654^25^	AY833325^3^	GU440517^27^	AY833368^3^

The source of the GenBank sequence ID used to represent each taxon at each molecular locus is indicated with superscript as follows: 1, Kontula et al. (2003); 2, Yokoyama and Goto (2005); 3, Kinziger et al. (2005); 4, Hubert et al. (2008); 5, Kinziger and Wood (2003); 6, Fast et al. (2017); 7, Park et al. (2006); 8, Smith and Busby (2014); 9, Smith and Wheeler (2004); 10, April et al. (2011); 11, Espinasa et al. (2014); 12, Knope (2013); 13, Elz et al. (2013b); 14, Elz et al. (2013a); 15, Bergsten et al. (2014); 16, International Barcode of Life (2011); 17, Baumsteiger et al. (2014); 18, Tabata et al. (2016); 19, Buser and López (2015); 20, Mandic et al. (2009); 21, Mecklenburg et al. (2011); 22, Steinke et al. (2009); 23, Elz et al. (2012); 24, Ramon and Knope (2008); 25, Hastings and Burton (2008); 26, Crow et al. (2004); 27, Hastings and Burton (2010); 28, Yamazaki et al. (2013); 29, Kontula and Väinölä (2003). Asterisk (*) denotes molecular sequence data that were provided by Dr. Matthew Knope.

### Phylogenetic inference

We assembled previously published molecular sequence data for each of our target species, downloading each from the online database GenBank (Sayers et al. 2019; Table 1). The prevalence of the use of mitochondrial loci in previous studies (especially in the genus *Cottus*) vastly outweigh the use of nuclear loci. We therefore selected five mitochondrial loci that have been sequenced extensively in sculpins and have maximal coverage among our targeted taxa: the small ribosomal subunit (12 s), a portion of the large ribosomal subunit (16Sar-br), ATPase 8 and 6 genes, cytochrome c oxidase subunit 1 (COI), and cytochrome b (cytb) (Table 1). The shortcomings of mitochondrial loci for use in inferring phylogenetic relationships are well known (e.g., Ballard and Whitlock 2004), but for many of the taxa included herein, there are no alternatives currently available. When possible, we included multiple (up to 10) sequences per species for each molecular locus.

For the protein-coding regions COI and cytb, we set the reading frame to minimize stop codons and translated each sequence from nucleotide triplets to the amino acids for which each encodes using Mesquite v.3.51 (Maddison and Maddison 2016). We aligned the resulting protein sequences using MUSCLE v.3.8.31 (Edgar 2004) using the default parameter settings and enforced this alignment on the original nucleotide sequences within Mesquite. The complex structure of 12 s, 16Sar-br, and ATPase 8 and 6 precluded unambiguous sequence alignment by protein translation, so instead we aligned these regions simply by nucleotide using MUSCLE (again using default parameter settings) within Mesquite. For each MSA, we visually assessed the robustness of the alignment. For the protein-coding loci, we checked for gaps and stop codons. For each of the remaining loci, we checked to ensure that the conserved areas of each locus aligned well, and that there were no excessive regions of ambiguous alignment. For all MSAs, we trimmed the sequences at the 5′ and 3′ end to eliminate missing data sites.

We conducted a maximum likelihood (ML) phylogenetic inference of the multiple sequence alignment (MSA) of each locus using RAxML v.8.2.10 (Stamatakis 2006, 2014) to test the species identification of each nucleotide sequence. We analyzed each locus separately (i.e., we inferred individual gene trees) and treated the locus as a single partition. For each of these analyses, we specified the rapid bootstrapping algorithm (Stamatakis et al. 2008) and applied the general time reversible model of molecular evolution with a gamma distribution of rate variation and invariable sites (GTR+I + Γ). For the ML phylogeny of each locus, we conducted a bootstrap analysis with 1000 iterations to assess the strength of the phylogenetic signal for each node therein.

We used the results of the ML gene tree analyses to verify the species identification of each sequence by ensuring that it (1) formed a clade with conspecific sequences and/or (2) followed expected phylogenetic placement based on previous studies. Following verification, we selected a single representative of each species for each locus. We did this not only to dramatically decrease analysis time for the final phylogenetic inference, but also because some loci in our dataset (i.e., COI, cytb) have large numbers of sequences available for each of our targeted species, while other loci do not. We concatenated these trimmed MSA datasets using Mesquite and partitioned COI, and cytb by codon position and treated 12 s, 16Sar-br, and ATPase 8 and 6 each as a single partition, resulting in a total of nine partitions. We used this dataset to infer a phylogenetic hypothesis of our target species using Bayesian inference (Drummond et al. 2002), conducted in BEAST v2.4.5 (Bouckaert et al. 2014) using the BEAGLE computing library (Ayres et al. 2012) on the CIPRES Science Gateway computing cluster (Miller et al. 2010).

For each partition in our dataset, we treated the model of molecular evolution as a parameter to be explored by the Markov chain Monte Carlo (MCMC) in our analysis using the bModelTest package (Bouckaert 2015) implemented in BEAST. We allowed the MCMC to consider all reversible models in that parameter space. We modeled the rate of molecular evolution as a lognormal relaxed clock (Drummond et al. 2006), unlinked across all loci. We specified a single tree model for our dataset with a birth–death speciation prior (Gernhard 2008) and specified *H.decagrammus* as the outgroup by constraining the tree to include all other species in our dataset as a monophyletic group. We specified a starting tree that contains *H. decagrammus* as sister to a polytomy containing all remaining taxa in our dataset. We performed four independent MCMC runs of 500 million generations each, sampled every 25,000 generations. We assessed convergence of our MCMC runs and determined the appropriate number of generations to discard as burn in using Tracer v.1.6.0 (Rambaut et al. 2014). We discarded burn in and combined the tree files using LogCombiner v.2.4.8 (Drummond and Rambaut 2007). We used TreeAnnotator v2.4.5 (Drummond and Rambaut 2007) to determine the maximum clade credibility (MCC) tree and posterior probability values of the nodes therein.

### Diet and habitat categorization

We reviewed published diet data and categorized the primary diet of each species in two ways: (1) using commonly-accepted diet categories such as insectivore, molluscivore, etc., according to whether one particular prey type (e.g., insect larvae, snails) occurred in frequencies or volumes >50% of the total diet; and (2) using the novel prey categories synthesized from the known diets of our sculpin taxa (described below). These diet categories will be referred to as “coarse” and “synthetic,” respectively, throughout the text below.

To infer our synthetic prey categories, we recorded the importance (e.g., percent volume) of prey items in the diet of each sculpin species from published accounts and records (Table 2). For species with multiple available diet studies and/or for diet studies partitioned by distinct geographic regions (e.g., water bodies) or temporal periods (e.g., seasons), we used the mean value of the importance of each unique component of diet (i.e., each prey item) across all studies and/or partitions. Where possible, we used diet data only from adults. For diet descriptions that did not specify importance, we assigned equal importance to all prey items included in the description.

**Table 2:** Character states of habitat, synthetic diet category, and coarse diet category; percent importance of prey items from each synthetic diet category in the diet; and diet data references

				% Importance in diet							
Species	Habitat	Synthetic diet category	Coarse diet category	Vermes	Stationary benthic items	Tentacles and appendages	Benthic arthropods	Pelagic arthropods	Squishy swimmers	References	NOAA
*Abyssocottus korotneffi*	Freshwater	Benthic arthropods	Invertivore	0.00	0.00	0.00	0.96	0.00	0.00	Sideleva and Mekhanikova (1990); Sitnikova et al. (2017)	
*Artedius fenestralis*	Marine	Benthic arthropods	Omnivore	0.19	0.12	0.00	0.64	0.00	0.00	Miller et al. (1980); Norton (1995)	
*Blepsias cirrhosus*	Marine	Benthic arthropods	Planktivore	0.00	0.00	0.00	0.59	0.39	0.00	Miller et al. (1980)	
*Chitonotus pugetensis*	Marine	Benthic arthropods	Invertivore	0.08	0.00	0.00	0.84	0.01	0.07	Miller et al. (1980); Norton (1995)*	
*Clinocottus acuticeps*	Marine	Benthic arthropods	Invertivore	0.00	0.00	0.00	0.95	0.06	0.00	Miller et al. (1980)	
*Clinocottus analis*	Marine	Benthic arthropods	Omnivore	0.24	0.17	0.01	0.49	0.02	0.01	Yoshiyama et al. (1986)	
*Clinocottus embryum*	Marine	Tentacles and appendages	Omnivore	0.09	0.09	0.39	0.39	0.05	0.00	Simenstad and Nakatani (1977); Miller et al. (1980)	
*Clinocottus globiceps*	Marine	Stationary benthic items	Omnivore	0.02	0.74	0.02	0.01	0.13	0.00	Miller et al. (1980); Norton (1995)	
*Clinocottus recalvus*	Marine	Stationary benthic items	Omnivore	0.00	0.43	0.07	0.14	0.36	0.00	Johnston (1954)	
*Comephorus dybowskii*	Freshwater	Pelagic arthropods	Planktivore	0.00	0.00	0.00	0.00	0.93	0.08	Sideleva (1996); Miyasaka et al. (2006)	
*Cottocomephorus grewingki*	Freshwater	Pelagic arthropods	Planktivore	0.00	0.00	0.00	0.00	0.80	0.20	Yoshii et al. (1999)	
*Cottus aleuticus*	Freshwater	Benthic arthropods	Insectivore	0.00	0.05	0.00	0.90	0.00	0.05	Scott and Crossman (1973); McPhail (2007)	
*Cottus asper*	Freshwater	Benthic arthropods	Insectivore	0.03	0.00	0.00	0.90	0.00	0.08	Northcote (1954); Patten (1962); Scott and Crossman (1973); Berejikian (1995); McPhail (2007)	
*Cottus asperrimus*	Freshwater	Benthic arthropods	Insectivore	0.02	0.19	0.00	0.71	0.06	0.00	Daniels and Moyle (1978)	
*Cottus baileyi*	Freshwater	Benthic arthropods	Insectivore	0.00	0.00	0.00	1.00	0.00	0.00	Novak and Estes (1974)	
*Cottus bairdii*	Freshwater	Benthic arthropods	Insectivore	0.03	0.05	0.00	0.90	0.00	0.03	Ricker (1934); Daiber (1956); Scott and Crossman (1973)	
*Cottus beldingii*	Freshwater	Stationary benthic items	Omnivore	0.20	0.67	0.00	0.13	0.01	0.05	Ebert and Summerfelt (1969); Moyle (1976)	
*Cottus carolinae*	Freshwater	Benthic arthropods	Insectivore	0.00	0.00	0.00	0.87	0.00	0.12	Etnier and Starnes (1993); Phillips and Kilambi (1996)	
*Cottus cognatus*	Freshwater	Benthic arthropods	Omnivore	0.00	0.00	0.00	0.97	0.00	0.02	Van Vliet (1964); Scott and Crossman (1973)	
*Cottus confusus*	Freshwater	Benthic arthropods	Insectivore	0.00	0.00	0.00	0.77	0.00	0.23	Johnson et al. (1983)	
*Cottus extensus*	Freshwater	Pelagic arthropods	Invertivore	0.00	0.00	0.00	0.00	1.00	0.00	Neverman and Wurtsbaugh (1994)	
*Cottus gobio*	Freshwater	Benthic arthropods	Insectivore	0.08	0.00	0.00	0.62	0.00	0.00	Mills and Mann (1983)	
*Cottus gulosus*	Freshwater	Benthic arthropods	Insectivore	0.10	0.00	0.00	0.85	0.00	0.05	Moyle (1976); Baltz et al. (1982)	
*Cottus hubbsi*	Freshwater	Benthic arthropods	Insectivore	0.00	0.00	0.00	0.90	0.00	0.10	McPhail (2007)	
*Cottus klamathensis*	Freshwater	Benthic arthropods	Insectivore	0.00	0.00	0.00	1.00	0.00	0.00	Rutter (1908); Robins and Miller (1957); Bond (1963); Moyle (1976)	
*Cottus leiopomus*	Freshwater	Benthic arthropods	Insectivore	0.00	0.00	0.00	1.00	0.00	0.00	Merkley and Griffith (1993)	
*Cottus perplexus*	Freshwater	Benthic arthropods	Piscivore	0.00	0.03	0.00	0.93	0.00	0.05	Phillips and Claire (1966); Moyle (1976)	
*Cottus pitensis*	Freshwater	Benthic arthropods	Insectivore	0.00	0.00	0.00	1.00	0.00	0.00	Li and Moyle (1976); Moyle (1976)	
*Cottus poecilopus*	Freshwater	Benthic arthropods	Omnivore	0.05	0.00	0.00	0.90	0.00	0.05	Gabler and Amundsen (1999); Holmen et al. (2003); Kotusz et al. (2004)	
*Cottus pollux*	Freshwater	Benthic arthropods	Insectivore	0.00	0.00	0.00	1.00	0.00	0.00	Natsumeda et al. (2012)	
*Cottus rhotheus*	Freshwater	Squishy swimmers	Insectivore	0.00	0.00	0.00	0.50	0.00	0.50	Scott and Crossman (1973)	
*Cottus ricei*	Freshwater	Benthic arthropods	Insectivore	0.00	0.00	0.00	1.00	0.00	0.00	Scott and Crossman (1973)	
*Dasycottus setiger*	Marine	Benthic arthropods	Invertivore	0.04	0.00	0.00	0.72	0.10	0.10	Jewett et al. (1989); Norton (1995)	NOAA
*Enophrys bison*	Marine	Benthic arthropods	Omnivore	0.11	0.31	0.00	0.54	0.00	0.00	Hart (1973); Miller et al. (1980); Norton (1995)	
*Gymnocanthus galeatus*	Marine	Benthic arthropods	Omnivore	0.27	0.00	0.08	0.38	0.05	0.09	Simenstad and Nakatani (1977); Tokranov (1985); Napazakov and Chuchukalo (2003)	
*Hemilepidotus jordani*	Marine	Benthic arthropods	Omnivore	0.10	0.05	0.02	0.53	0.00	0.22	Brodeur and Livingston (1988)	NOAA
*Hemilepidotus zapus*	Marine	Tentacles and appendages	Invertivore	0.20	0.04	0.44	0.11	0.00	0.08	Tokranov et al. (2003); Tokranov and Orlov (2007)	
*Hemitripterus bolini*	Marine	Squishy swimmers	Piscivore	0.00	0.00	0.00	0.00	0.00	1.00	Brodeur and Livingston (1988); TenBrink and Hutchinson (2009)	NOAA
*Hexagrammos decagrammus*	Marine	Benthic arthropods	Omnivore	0.13	0.10	0.00	0.52	0.00	0.24	Miller et al. (1980)	NOAA
*Icelinus filamentosus*	Marine	Benthic arthropods	Invertivore	0.00	0.00	0.00	1.00	0.00	0.00	Hart (1973)	
*Icelus spiniger*	Marine	Benthic arthropods	Omnivore	0.00	0.00	0.00	0.87	0.00	0.11	Andriyashev (1954); Atkinson and Percy (1992)	NOAA
*Jordania zonope*	Marine	Benthic arthropods	Invertivore	0.19	0.00	0.00	0.51	0.22	0.00	Burge and Schultz (1973); Demetropoulos et al. (1990); Norton (1995)	
*Leptocottus armatus*	Marine	Benthic arthropods	Omnivore	0.00	0.00	0.00	0.65	0.00	0.35	Miller et al. (1980); Norton (1995)	
*Microcottus sellaris*	Marine	Benthic arthropods	Invertivore	0.03	0.00	0.00	0.75	0.09	0.12	Maksimenkov (1996)	
*Myoxocephalus polyacanthocephalus*	Marine	Benthic arthropods	Piscivore	0.00	0.00	0.00	0.77	0.02	0.15	Simenstad and Nakatani (1977); Miller et al. (1980); Brodeur and Livingston (1988); Norton (1995)	
*Oligocottus maculosus*	Marine	Benthic arthropods	Invertivore	0.03	0.00	0.00	0.88	0.00	0.00	Miller et al. (1980); Norton (1995)	
*Oligocottus rimensis*	Marine	Benthic arthropods	Invertivore	0.00	0.00	0.00	0.79	0.21	0.00	Miller et al. (1980); Grossman (1986)	
*Oligocottus snyderi*	Marine	Benthic arthropods	Invertivore	0.14	0.00	0.00	0.67	0.03	0.00	Miller et al. (1980); Yoshiyama (1980); Freeman et al. (1985); Norton (1995)	
*Orthonopias triacis*	Marine	Benthic arthropods	Invertivore	0.38	0.00	0.00	0.62	0.00	0.00	Burge and Schultz (1973); Norton (1995); Snook (1997)	
*Porocottus camtschaticus*	Marine	Vermes	Invertivore	0.82	0.09	0.00	0.09	0.00	0.00	Saveliev and Kolpakov (2016)	
*Psychrolutes phrictus*	Marine	Benthic arthropods	Omnivore	0.01	0.00	0.04	0.94	0.00	0.00	Eschmeyer et al. (1983)	NOAA
*Rhamphocottus richardsonii*	Marine	Benthic arthropods	Invertivore	0.07	0.00	0.00	0.62	0.22	0.00	Hart (1973); Eschmeyer et al. (1983); Norton (1995)	
*Scorpaenichthys marmoratus*	Marine	Squishy swimmers	Omnivore	0.04	0.00	0.00	0.37	0.00	0.58	Burge and Schultz (1973); Hart (1973); Norton (1995)	
*Triglops scepticus*	Marine	Pelagic arthropods	Omnivore	0.04	0.00	0.00	0.11	0.70	0.12	Atkinson and Percy (1992)	NOAA

Diet data from NOAA was provided by the National Oceanic Atmospheric Administration National Marine Fisheries Service, Alaska Fisheries Science Center, and Resource Ecology and Ecosystem Modeling Program. * Diet data for *C. pugetensis* from Norton (1995) were adjusted to account for what appears to be a decimal place error: the percentage of shrimp in the diet was interpreted to be 30% rather than 3%.

We recorded all unique prey items found among all sculpin species and coded the presence/absence of 25 functional traits for each prey item (Table 3). We constructed a matrix of the Euclidean distances of each prey item based on their functional attributes and used Ward’s linkage method on the distance matrix to cluster the prey items. We plotted the within groups sum of squares for each potential number of clusters and used the inflection point of the graph (i.e., a broken-stick style assessment) to determine the appropriate number of synthetic prey categories. We assigned the primary diet of each sculpin species to one of the synthetic prey categories by calculating the importance of constituent prey items in the diet of a given sculpin species, then categorizing the primary diet of said species as whichever category encompassed the highest importance of prey items. We calculated the importance of each diet category for a given species by summing the importance of each constituent prey item for each diet category. We classified the primary diet of each sculpin species as the diet category containing the highest sum of prey item importance.

**Table 3:** Functional traits for all unique prey items recorded in the diet of sculpin taxa included in this study

	Function traits																								
Prey taxon	Body covered by chitinous exoskeleton	Body surrounded by calcareous shell	Internal bony skeleton	Motile (0) or sessile (1)?	Demersal?	Pelagic?	Fossorial	Worm-like body shape	Segmentation of body	Animal?	Capable of swimming	Fast swimmer	High lipid content	Difficult to digest (chitin, cellulose)	Defensive spine(s)	Defensive pincers	Prey taxon is herbivore	Prey taxon is detritivore	Prey taxon is carnivore	Prey taxon is planktivore	Multiple appendages	Complex eyes	Cephalization	Substrate gripping ability	Stinging tentacles
Algae and plant matter	0	0	0	1	0	0	0	0	0	0	0	0	0	1	0	0	0	0	0	0	0	0	0	1	0
Anemone	0	0	0	1	0	0	0	1	0	1	0	0	0	0	0	0	0	0	1	1	1	0	0	1	1
Barnacle cirri	1	0	0	1	0	0	0	0	1	1	0	0	0	1	0	0	0	0	1	1	1	0	1	1	0
Bivalvia	0	1	0	1	0	0	1	0	0	1	0	0	0	0	0	0	0	1	0	1	0	0	0	1	0
Copepoda	1	0	0	0	1	1	0	0	1	1	1	0	0	1	0	0	1	0	0	1	1	1	1	0	0
Crab	1	0	0	0	0	0	0	0	1	1	0	0	0	1	0	1	1	1	1	0	1	1	1	1	0
Crayfish	1	0	0	0	0	0	0	0	1	1	1	1	0	1	0	1	1	1	1	0	1	1	1	1	0
Ctenophora	0	0	0	0	0	1	0	0	0	1	1	0	0	0	0	0	0	0	1	1	1	0	0	0	1
Cumacea	1	0	0	0	1	0	1	0	1	1	1	0	0	1	0	0	1	1	0	1	1	1	1	0	0
Detritus	0	0	0	1	0	0	0	0	0	0	0	0	0	0	0	0	0	0	0	0	0	0	0	0	0
Eggs	1	0	0	1	0	0	0	0	0	1	0	0	1	0	0	0	0	0	0	0	0	0	0	0	0
Euphausiidae	1	0	0	0	1	1	0	0	1	1	1	0	1	1	1	0	1	0	0	0	1	1	1	0	0
Fishes	0	0	1	0	1	1	0	0	0	1	1	1	1	0	1	0	0	0	1	1	1	1	1	0	0
Gammaridae	1	0	0	0	0	0	0	0	1	1	1	0	0	1	0	0	1	1	0	1	1	1	1	1	0
Gastropoda	0	1	0	0	0	0	0	0	0	1	0	0	0	0	0	0	1	1	1	0	0	1	1	1	0
Hermit crab	1	1	0	0	0	0	0	0	1	1	0	0	0	1	0	1	1	1	1	0	1	1	1	1	0
Insecta	1	0	0	0	0	0	0	0	1	1	1	0	0	1	0	0	1	1	0	0	1	1	1	1	0
Isopoda	1	0	0	0	0	0	0	0	1	1	0	0	0	1	0	0	1	1	0	0	1	1	1	1	0
Larval fishes	0	0	1	0	0	1	0	0	0	1	1	0	0	0	1	0	0	0	1	1	1	1	1	0	0
Leech (Hirudinea)	0	0	0	0	0	0	0	1	0	1	1	0	0	0	0	0	0	0	1	0	0	0	1	0	0
Mysidae	1	0	0	0	1	1	0	0	1	1	1	0	1	1	0	0	1	0	0	1	1	1	1	0	0
Octopus	0	0	0	0	0	0	0	0	0	1	1	1	0	0	0	0	0	0	1	0	1	1	1	1	0
Oligochaeta	0	0	0	1	0	0	1	1	1	1	0	0	0	0	0	0	0	1	0	0	0	0	1	0	0
Ostracoda	1	1	0	0	1	1	0	0	1	1	1	0	0	1	0	0	1	0	1	1	1	1	1	0	0
Pelagic amphipod	1	0	0	0	0	1	0	0	1	1	1	0	1	1	0	0	1	0	0	1	1	1	1	0	0
Planaria	0	0	0	0	0	0	0	1	0	1	0	0	0	0	0	0	0	1	0	1	0	1	1	0	0
Polychaete annelid	0	0	0	0	0	0	1	1	1	1	0	0	0	0	0	1	0	0	1	0	0	0	1	0	0
Pandelid shrimp	1	0	0	0	0	0	0	0	1	1	1	1	0	1	1	1	0	1	1	0	1	1	1	1	0
Sipuncula	0	0	0	1	0	0	1	1	0	1	0	0	0	0	0	0	0	1	0	0	0	0	1	0	0

Unless stated otherwise, character states are recorded as presence (1)/absence (0) or true (1)/false (0).

Finally, we categorized the primary habitat of each sculpin species as either “freshwater” or “marine” by reviewing species accounts in the literature (Bolin 1944; Mecklenburg et al. 2002; Goto et al. 2015; Kells et al. 2016; Nelson et al. 2016). There are many sculpin taxa with the ability to live in both marine and freshwater habitats, so for the purposes of this study, we assigned the primary habitat as that in which most populations of a given species spend the majority of their life history. We conducted an ancestral state reconstruction (ASR) of both habitat and the synthetic diet categories in Mesquite. We specified the Mk1 model of discrete trait evolution of the characters across the MCC phylogeny inferred herein.

### Morphological measurements

We acquired specimens to represent each of the 54 species included in this study from museum collections (Table 4). While there is considerable variation in the maximum size recorded for species in our taxon sample, we selected individuals of adult (i.e., sexually mature) size where such data are known. Thereby, we sought to avoid mischaracterizing the morphology of a given species, especially for species with known shifts in habitat between juvenile and adult life stages (e.g., Brandt 1986; Ruzycki and Wurtsbaugh 1999). Likewise, while some species of sculpin show intraspecific variability in some morphological traits associated to feeding (and thus pertinent to the present study), these traits do not show overlap across species (Kerfoot and Schaefer 2006). We µCT scanned the specimens in batches using the 1173 Bruker Skyscan µCT system at the Karl Liem Bioimaging Center at Friday Harbor Laboratories (Friday Harbor, WA). We used scanning parameters ranging from 60 to 75 kV and 100 to 133 µA, and resolution from 18.1 to 54.7 µm (voxel size). We used a 1 mm aluminum filter on all scans. We reconstructed the resulting image stacks using NRecon (Bruker microCT, Kontich, Belgium, 2016) and isolated individual fish from each batch in DataViewer 2.1 (Bruker, Kontich, Belgium, 2010). We converted these image stacks to DICOM file format for viewing and segmentation in the computer program Horos v2.0.1 (The Horos Project, 2015; http://www.horosproject.org/) and CTVox 2.7 software (Bruker Corp., Billerica, MA).

**Table 4:** Museum catalog number, X-ray source voltage in kilovolts (kV), X-ray source intensity in micro-amperes (µA), three-dimensional pixel (voxel) size in microns for reconstructed image, standard length (SL) in millimeters (mm) of the specimen, and unique identification number of the tomographic data on MorphoSource (www.morphosource.org) for each specimen used in this study

Taxon	Catalog Number	kV	µ**A**	Voxel size	SL (mm)	MorphoSourceID
*Abyssocottus korotneffi*	USNM 362049	63	119	28	53.01	M15541-28601
*Artedius fenestralis*	OSIC 09206	75	100	50	65.00	M15616-33119
*Blepsias cirrhosus*	UW 025364	60	133	49.7	73.00	M15741-29180
*Chitonotus pugetensis*	OSIC 14872	75	100	50	75.66	M15324-28851
*Clinocottus* (*Oxycottus*) *acuticeps*	UAM 47713	70	114	54.7	41.31	M28728-55222
*Clinocottus* (*Clinocottus*) *analis*	OSIC 000914	70	114	54.7	43.59	M28227-54617
*Clinocottus* (*Blennicottus*) *embryum*	UAM 47704	70	114	54.7	40.11	M28270-73055
*Clinocottus* (*Blennicottus*) *globiceps*	OSIC 000275	70	114	54.7	44.14	M27980-73058
*Clinocottus* (*Blennicottus*) *recalvus*	SIO 249-55	70	114	54.7	47.00	M28220-73059
*Comephorus dybowskii*	OSIC 004306	60	110	18.1	80.46	M15421-28270
*Cottocomephorus grewingki*	OSIC 04244	60	133	49.7	97.15	M15433-28292
*Cottus aleuticus*	OSIC 016040	60	133	49.7	91.33	M15714-29114
*Cottus asper*	OSIC 013876	60	133	49.7	64.52	M15632-28901
*Cottus asperrimus*	OSIC 011018	60	133	49.7	69.46	M15666-28992
*Cottus baileyi*	CAS 226476	70	114	35.5	66.07	M15598-28806
*Cottus bairdii*	OSIC 05590	60	133	49.7	70.31	M15668-28997
*Cottus beldingii*	OSIC 19179	60	133	49.7	84.19	M15695-29060
*Cottus carolinae*	OSIC 00259	60	133	49.7	58.96	M15601-31974
*Cottus cognatus*	OSIC 08356	60	133	49.7	88.22	M15710-29106
*Cottus confusus*	OSIC 00596	60	133	49.7	83.28	M16458-30611
*Cottus extensus*	OSIC 06579	60	110	24.9	57.48	M15582-28769
*Cottus gobio*	OSIC 01759	60	133	49.7	44.10	M15436-28299
*Cottus gulosus*	OSIC 10534	60	133	49.7	68.51	M15642-28924
*Cottus hubbsi*	OSIC 18845	60	133	49.7	70.46	M15671-33118
*Cottus klamathensis*	OSIC 18295	60	110	24.9	58.41	M15584-28771
*Cottus leiopomus*	OSIC 05589	60	133	49.7	73.92	M15742-29183
*Cottus perplexus*	OSIC 09251	60	110	24.9	56.81	M15586-28775
*Cottus pitensis*	OSIC 06487	60	133	49.7	52.34	M15516-28538
*Cottus poecilopus*	UW 044760	67	119	29.1	73.86	M16942-31476
*Cottus pollux*	UW 011690	60	133	49.7	72.00	M15739-29176
*Cottus rhotheus*	OSIC 18849	60	133	49.7	86.96	M15707-29100
*Cottus ricei*	UW 03368	60	133	49.7	43.00	M15474-28409
*Dasycottus setiger*	OSIC 07086	60	133	49.7	66.99	M15633-28903
*Enophrys bison*	OSIC 07445	60	110	24.9	57.00	M15587-28777
*Gymnocanthus galeatus*	UW 026347	60	133	49.7	78.00	M15763-29249
*Hemilepidotus jordani*	OSIC 03421	60	133	49.7	93.52	M15635-28908
*Hemilepidotus zapus*	UW 111999	60	133	49.7	75.00	M15757-29236
*Hemitripterus bolini*	OSIC 15252	60	133	49.7	75.32	M15760-29242
*Hexagrammos decagrammus*	OSIC 00274	60	133	49.7	62.95	M15619-28869
*Icelinus filamentosus*	UW 04863	60	133	49.7	67.00	M15763-29249
*Icelus spiniger*	OSIC 08761	60	133	49.7	63.32	M15622-28877
*Jordania zonope*	OSIC 07015	60	133	49.7	67.74	M15649-28940
*Leptocottus armatus*	OSIC 00811	60	133	49.7	59.95	M15602-28824
*Microcottus sellaris*	OSIC 08697	60	133	49.7	61.94	M15623-28879
*Myoxocephalus polyacanthocephalus*	UW 02690	60	133	49.7	74.00	M15326-29189
*Oligocottus maculosus*	OSIC 000287	70	114	54.7	42.15	M28053-54262
*Oligocottus rimensis*	SIO 67-151	70	114	54.7	43.75	M28226-54609
*Oligocottus snyderi*	OSIC 004366	70	114	54.7	39.10	M40466-73063
*Orthonopias triacis*	OSIC 08137	75	100	50	63.50	M28062-54285
*Porocottus camtschaticus*	UW 042699	60	133	49.7	40.00	M15482-28433
*Psychrolutes phrictus*	OSIC 13541	60	133	49.7	115.56	M15652-28948
*Rhamphocottus richardsonii*	UW 016400	60	133	49.7	40.00	M15471-28398
*Scorpaenichthys marmoratus*	OSIC 03423	60	133	49.7	59.36	M15472-28403
*Triglops scepticus*	OSIC 17469	60	133	49.7	86.39	M15698-29067

Museum abbreviations follow Sabaj (2016): University of Alaska Museum (UAM), Oregon State Ichthyology Collection (OSIC), Scripps Institution of Oceanography (SIO), Smithsonian National Museum of Natural History (USNM), California Academy of Science (CAS), and University of Washington’s Burke Museum of Natural History (UW).

**Fig. 1:**
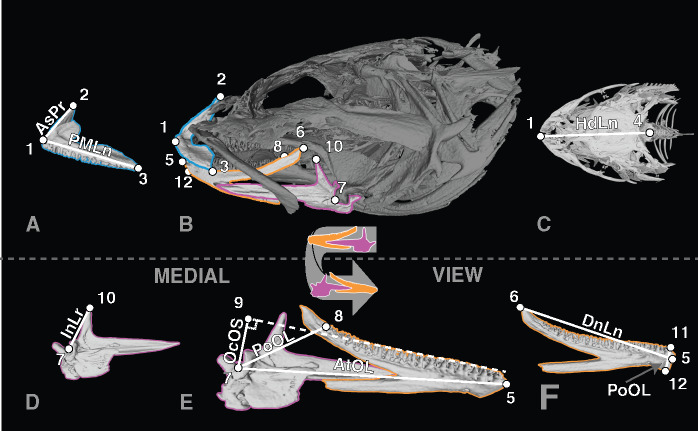
Biomechanical jaw measurements used to capture the functional morphology of the feeding apparatus of sculpins in our study. Each landmark (LM) is defined as follows: LM1, anteriormost point of the premaxilla; LM2, postero-dorsal most point of the ascending process of the premaxilla; LM3, posteriormost point on the premaxilla; LM4, postero-dorsal most point of the supraoccipital; LM5, anteriormost point of the dentary; LM6, postero-dorsal most point of the dorsal margin of the dentary; LM7, lowest point (trough) in the fossa of the angular where it articulates with the condyle of the quadrate to form the quadroangular articulation; LM8, position on the dentary at the base of the posteriormost tooth; LM9, point where a tangent line from the tooth row is closest to point 7; LM10, dorsalmost point of the ascending process of the angular; LM11, dorsal most point of dentary at the symphysis; LM12, ventral most point of dentary at the symphysis. These landmarks are also annotated onto a 3D model of the skull, available at https://skfb.ly/6HsWW. Measurements are defined as follows: ascending process length (AsPr), LM1–LM2; premaxilla length (PMLn), LM1–LM3; head length (HdLn), LM1–LM4; dentary length (DnLn), LM5–LM6; anterior out-lever (AtOL), LM5–LM7; posterior out-lever (PoOL), LM7–LM8; occlusal offset (ArOS), LM7–LM9; in-lever (InLr), LM7–LM10; mandible symphysis height (MaSH), LM11–LM12. The landmarks and measurements are illustrated on a micro-CT reconstruction of the cranial bones from a specimen of *Cottus rhotheus* (Oregon State Ichthyology Collection 18849, 86.96 mm SL). **A**) The isolated left premaxilla in lateral view. **B**) The fully-articulated cranium in lateral view with the premaxilla highlighted in blue, the dentary in orange, and the angular–articular in purple. **C**) The cranium in dorsal view. **D**) The isolated left angular–articular in medial view. **E**) The isolated left lower jaw in medial view with the dentary highlighted in orange and angular–articular in purple. **F**) The isolated left dentary in medial view. Rotatable 3D model of this illustrated skull available on SketchFab: https://skfb.ly/6HsWW.

We used the line tool in the 3D-MPR function in Horos to measure a series of morphological traits that have been used in previous studies to capture important aspects of the feeding mechanism in fishes across broad dietary guilds (see Fig. 1; Hulsey and De Leon 2005; Anderson 2009; Anderson et al. 2013; Arbour and López-Fernández 2013, 2014; Kolmann et al. 2018). From these measures, we calculated the following characters: (1) anterior and (2) posterior closing mechanical advantage of the jaws, i.e., measures of jaw leverage and mouth-closing velocity; (3) occlusal offset, a proxy for how the teeth are brought into occlusion, varying from scissor-like action to precise occlusion; (4) tooth aspect ratio, a measure of the degree to which teeth are either squat or cuspidate; (5) symphyseal height, a measure of robustness where the rami of the upper and lower jaw halves meet; (6) relative head length, a measure of the length of the head relative to standard body length; and (7) the ratio of ascending process height to premaxillary length, a proxy for jaw protrusion. Each of these seven characters is a ratio (see descriptions in Fig. 1 caption) and so provides a relativized value that is robust to differences in the absolute length of each specimen. Together, these traits describe how differing fish anatomies are built to, for example, capture elusive prey, shear or crush shelled prey, hold struggling prey, or protrude jaws away from the cranium in order to seize prey.

### Relationship of diet and morphology

We visually assessed the normality of our data using quantile–quantile (qq) plots of each of our calculated variables and standardized these variables with a *z*-transformation using basic functions in the R statistical environment (R Core Team 2017). We visualized the morphological variance in our dataset by performing a principle component analysis (PCA) and overlaying the phylogenetic relationships of our taxa using the phylomorphospace approach (Sidlauskas 2008) with functions from the R package “geomorph v3.0.4” (Adams and Otárola-Castillo 2013; Adams et al. 2016). We used the broken stick method (Frontier 1976; Jackson 1993; Legendre and Legendre 2012) to select a subset of PC axes that each account for more variance than would be expected by chance using functions from the R package “vegan v2.4.3” (Oksanen et al. 2017). We used this subset of PC axes only to visualize the distribution of species in phylomorphospace. For all statistical tests, we used the *z*-transformed values of the seven morphological characters mentioned above, which are described and illustrated in Fig. 1.

We tested for differences in the average value of our seven morphological characters of the freshwater vs. saltwater species as well as among the species constituting each of our diet guilds using phylogenetic multivariate analysis of variance (MANOVA) with functions from the R package “GEIGER v 2.0.6” (Harmon et al. 2008). We tested for differences among diet guilds twice: once using the coarse categories and once using the synthetic diet categories. For each phylogenetic MANOVA, we used a Wilks test with 1000 replicates to simulate a *P*-value (Garland et al. 1993).

We tested for mismatch between the diet categorization of each sculpin and its morphology by performing a discriminant function analysis with functions from the R package “MASS v7.3.48” (Venablees and Ripley 2002) and comparing the posterior diet categorizations (i.e., based on morphology) with the original diet categorizations based on diet data. All pertinent data (measurements, diet categories, etc.) and an annotated R script that performs all operations conducted in R in this study is available in Supplementary Data S1.1–S1.4.

## Results

### Phylogenetic hypothesis

**Fig. 2:**
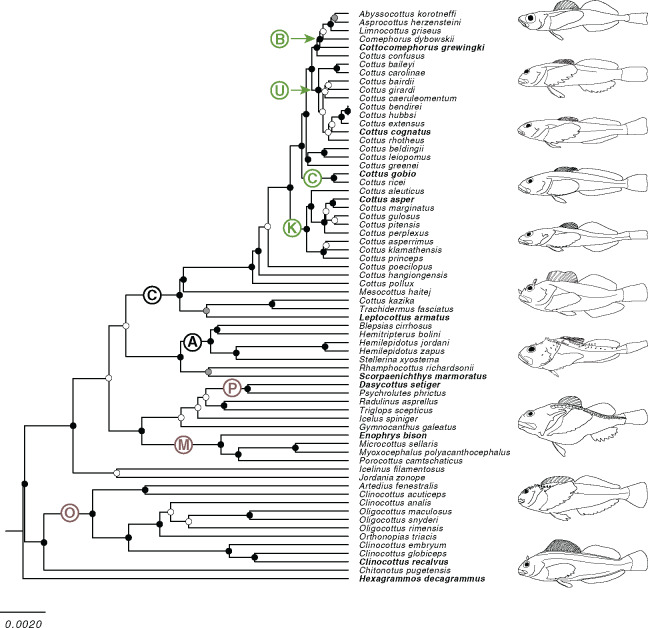
Phylogenetic hypothesis of 53 species in the superfamily Cottoidea and the outgroup taxon *Hexagrammos decagrammus*. This phylogeny is the maximum clade credibility tree from a Bayesian phylogenetic inference of previously published molecular sequence data. Bayesian posterior probabilities (BPPs) of each node were sampled from a posterior distribution of ∼70,000 trees and are represented as follows: black circles indicate BPP ≥ 0.95, gray circles indicate 0.95 > BPP ≥ 0.85, white circles indicate BPP < 0.85. Taxonomic groups are denoted with a circled letter on the branch leading to the most restrictive clade containing all members of a given group included in this study. Families are indicated with black text as follows: “C,” Cottidae; “A,” Agonidae. Subfamilies are indicated with brown text as follows: “M.” Myoxocephalinae; “P,” Psychrolutinae; “O,” Oligocottinae. Lineages within the genus *Cottus* are indicated as follows: “B,” Baikalian; “C,” Cottus; “K,” Cottopsis; “U,” Uranidae. Illustrated species are indicated by the taxon name in bold and appear in the same order from top to bottom: *Cottocomephorus grewingkii* (Oregon State Ichthyology Collection [OSIC] 4244, 97.15 mm SL), *Cottus cognatus* (OSIC 8359, 68.53 mm SL), *Cottus gobio* (OSIC 1759, 42.37 mm SL), *Cottus asper* (OSIC 5797 107.08 mm SL), *Leptocottus armatus* (OSIC 183330, 97.77 mm SL), *Scorpaenichthys marmoratus* (OSIC 8875, 161.53 mm SL), *Dasycottus setiger* (OSIC 6385, 138.16 mm SL), *Enophrys bison* (OSIC 11799, 233.12 mm SL), *Clinocottus recalvus* (OSIC 8134, 70.5 mm SL), and *Hexagrammos decagrammus* (OSIC 274, 62.95 mm SL).

The trimmed length of the MSA of each locus is as follows: 12 s, 726 base pairs (bp); 16Sar-br, 475 bp; ATPase 8 and 6, 829 bp; COI, 651 bp; cytb, 678 bp; for an aggregate total of 3359 aligned nucleotide sites. The trimmed nucleotide MSA, protein MSA (for protein-coding loci), and ML phylogeny for each locus is available in Supplementary Data S2.1–S2.5. The topology of our species tree is generally well-supported, especially at the level of taxonomic family and subfamily, as well as the four distinct lineages contained within the *Cottus* clade (Fig. 2). However, the support values are low at many basal nodes and the marine sculpins of the family Psychrolutidae failed to resolve as monophyletic. Rather, the MCC tree shows the subfamily Oligocottinae as sister to all other sculpins in our study, and the remaining families are intermixed with various psychrolutid taxa (Fig. 2).

### Diet and habitat

**Fig. 3:**
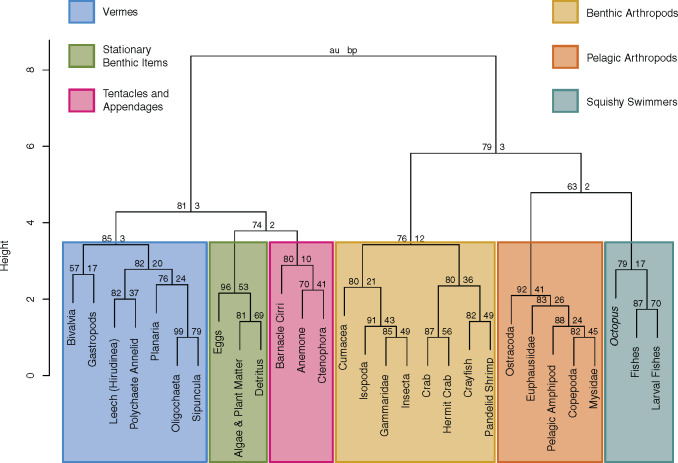
Dendogram of prey items clustered by their functional traits. Length on the vertical axis represents the distance between clusters. Synthetic prey categories are represented using color-coded boxes around the constituent prey items in each category. Support values of each cluster are represented at each node. The height of each branch is representative of the distance between each of its daughter lineages.

For the species, *Cottus extensus* and *Microcottus sellaris*, diet data are only available for the juvenile life-stage, but for all other species, we used only diet data from adults. We identified 29 unique prey items consumed by sculpins in our literature review (Tables 2 and 3). After clustering these items based on their functional traits, the within groups sum of squares analysis showed that six groups are appropriate to represent the prey items (see Supplementary Data S1.1–S1.4). These groups, and their constituent prey taxa, are presented in Fig. 3. For ease of reference, we have named each of these groups and will refer to them hereafter as follows: “Benthic Arthropods” is composed of the following prey items identified in sculpin diets from our literature review: Cumacea (Arthropoda: Crustacea), Isopoda (Arthropoda: Crustacea), Gammaridae (Arthropoda: Crustacea: Amphipoda), Insecta (Arthropoda: Hexapoda), crab (Arthropoda: Crustacea: Decapoda: brachyuran [Brachyura] and non-hermit anomuran [Anomura] crabs combined), hermit crabs (Arthropoda: Crustacea: Decapoda: Anomura), crayfish (Arthropoda: Crustacea: Decapoda: Astacoidea), and pandalid shrimp (Arthropoda: Crustacea: Decapoda: Pandalidae). Benthic Arthropods are the primary diet of most sculpins in our study (41/54 species, ∼76%). Each of the remaining groups made up the primary diet of <10% of the sculpin species in our study. “Pelagic Arthropods” (primary diet of 4/54 sculpin species, ∼7%) is composed of: Ostracoda (Arthropoda: Crustacea), Euphausiidae (Arthropoda: Crustacea: Euphausiacea), pelagic amphipods (Arthropoda: Crustacea: Amphipoda, pelagic taxa, e.g., *Macrohectopus*, grouped together), Copepoda (Arthropoda: Crustacea), and Mysidae (Arthropoda: Crustacea). “Stationary Benthic Items” (primary diet of 3/54 sculpin species, ∼6%) is composed of: eggs, algae and plant matter, and detritus. “Squishy Swimmers” (primary diet of 3/54 sculpin species, ∼6%) is composed of: *Octopus* (Mollusca: Cephalopoda), fishes (Vertebrata: Pisces, excluding larval forms), and larval fishes (Vertebrata: Pisces, including only larval forms). “Tentacles and Appendages” (primary diet of 2/54 sculpin species, ∼4%) is composed of: the cirri of barnacles (Arthropoda: Crustacea: Cirripedia), anemone (Cnidaria: Actiniaria), and Ctenophora. The final group of prey items identified in our cluster analysis is “Vermes” (primary diet of 1/54 sculpin species, ∼2%), which is composed of: Bivalvia (Mollusca), gastropods (Mollusca: Gastropoda), leeches (Annelida: Hirudinea), polychaete annelids (Annelida: Polychaeta), Planaria (Platyhelminthes: Planariidae), Oligochaeta (Annalida), and Sipuncula. The diet of each sculpin species is generally dominated by prey items from one category, with 48/54 species (∼89%) having a diet made up of >50% items from a single category (Table 2).

Although the synthetic diet categories are based solely on morphological or ecological similarity of the prey items, they appear to group prey items that co-appear in the diets of the sculpin species. For example, among species categorized as primarily feeding upon Benthic Arthropods, diet items from that category made up >70% of stomach contents in 28/48 species and made up >90% of stomach contents in 17/48 species. Only six species had a diet where no synthetic prey category described >50% of that sculpin’s diet. Thus, for most cottoid species, a single synthetic diet category based on functional traits of the prey items appears to be not only adequate for describing the diet of the sculpins, but in most cases describes the diet well. This is reasonable, as prey items such as “*Octopus*” and “fishes” (two of the prey items grouped by “Squishy Swimmers”; Fig. 3), while not closely related phylogenetically (i.e., they would not be grouped into a single coarse diet category) share many characteristics with which a would-be predator would have to grapple (e.g., acute vision, the ability to swim rapidly; Table 3), and it appears that octopuses and fishes are in fact eaten together (e.g., the diet of *Scorpaenichthys marmoratus*: see Norton 1995).

There is some agreement between the coarse and synthetic diet categorization schemes (Table 2), such that the synthetic category “Benthic Arthropods” mostly contains species categorized as “omnivore,” “invertivore,” or “insectivore.” The synthetic category “Squishy Swimmers” and the coarse diet category “piscivore” would presumably be highly similar, and in fact both contain three species. However, the two categories agree only on one of the species (*Hemitripterus bolini*).

**Fig. 4:**
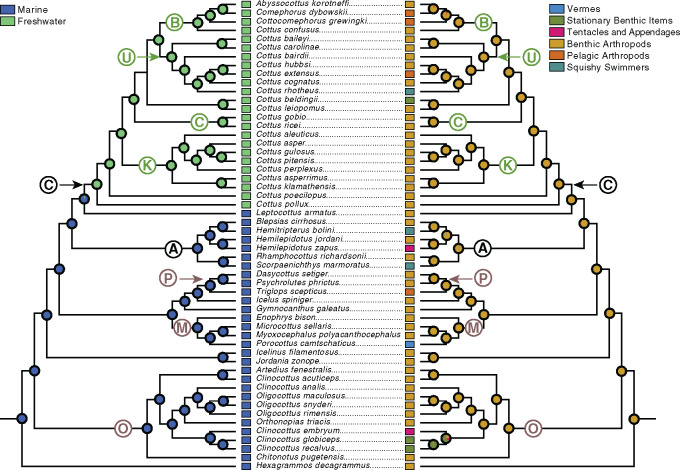
Ancestral state reconstruction of habitat (left side) and synthetic diet category (right side) on the MCC phylogeny depicted in Fig. 1. The pie chart at each node shows the proportional likelihood of each character state (indicated by color) at a given node. Taxonomic groups are indicated as in Fig. 1. The color of each synthetic prey category follows that of Fig. 3.

The ASR of habitat shows strong support for a single transition from marine to freshwater habitat in the branch separating the hypothetical most recent common ancestor (MRCA) of *Cottus* + *Leptocottus* and the MRCA of the members of the genus *Cottus* included in our study (Fig. 4). The ASR of our synthetic diet categories shows an evolutionary scenario where a diet primarily of Benthic Arthropods is the inferred ancestral state for all but one clade (Fig. 4). Given this scenario, diets primarily composed of anything other than Benthic Arthropods have evolved only relatively recently, independently, and at the level of species or genus. As such, shared diet guilds among taxa seem to have come about primarily through convergence. The exception in our dataset is the two closely related species, *Clinocottus* (*Blennicottus*) *globiceps* and *C.* (*B.*) *recalvus*, which both prey primarily upon Stationary Benthic Items.

### Functional morphospace/relationship of diet and morphology

The first principal component axis (PC1) captures ∼25% of the observed variance in our dataset and is dominated by the anterior mechanical advantage of the jaw (Character 1), such that high values of PC1 are associated with jaws that are relatively shortened in the antero-posterior dimension, while low values of PC1 are associated with jaws that are relatively elongate. The second principal component (PC2) captures ∼19% of observed variance and is dominated by posterior mechanical advantage of the jaw (Character 2), such that high values of PC2 are associated with high posterior mechanical advantage, while low values of PC2 are associated with low posterior mechanical advantage. The loadings and percent variances of all PC axes are summarized in Supplementary Data S3.

**Fig. 5:**
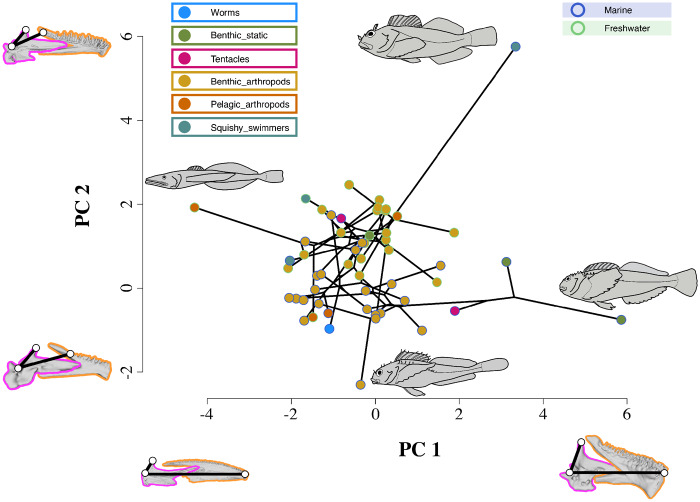
Phylomorphospace of the first two principal components of feeding functional morphology in freshwater and marine sculpin taxa. This figure is interactive when opened with Adobe Acrobat, and an interactive online version of this figure is hosted at: https://indd.adobe.com/view/527ec566-822f-4cd5-a572-130e8923f766. A non-interactive version of this figure is available in Supplementary Data S4. A colored dot (tip) represent each species included in this study. The interactive figure reveals the name of the species represented by each tip when the reader’s mouse hovers over. The lines connecting these dots represent the phylogenetic relationships of the taxa. The position of branching points (phylogenetic nodes) in the morphospace indicates the inferred state for a given hypothetical ancestor (see the “Materials and methods” section). Each tip is colored to show the habitat and synthetic diet category of the species that it represents. Habitat color is indicated by the outline color of the dot. Synthetic diet category is indicated by the fill color of the dot. The interactive figure shows convex hulls outlining the taxa that represent the character states for habitat and synthetic diet category. Clicking on the box for each character state reveals the representative convex hull. The morphological character with the greatest variance is illustrated for each principal component (PC) axis (see the “Results” section). The illustration shows the linear measurements associated with a given character from a medial perspective on the lower jaw of the taxon with the most extreme value of a given PC axis. The landmarks, linear measures, and color coding of the constituent bones of the lower jaw follow those in Fig. 1. The body shape of these taxa (i.e., those with the most extreme values of each PC axis) is illustrated next to their representative tip in morphospace as follows: PC 1 positive, *Clinocottus* (*Blennicottus*) *recalvus* (OSIC 8134, 70.5 mm SL); PC 2 negative, *Comephorus dybowskii* (OSIC 4306, 80.46 mm SL); PC 2 positive, *Scorpaenichthys marmoratus* (OSIC 8875, 161.53 mm SL); PC 3 negative *Clinocottus* (*Oxycottus*) *acuticeps* (UAM 47713, 47.16 mm SL).

The results of the broken stick analysis show that the first two PC axes account for more variance than would be expected by chance, so we used these two axes to illustrate the phylomorphospace, which is presented in Fig. 5 and Supplementary Data S4. There is substantial overlap of marine and freshwater species in the morphospace and the differences in average trait values therein are not statistically significant (*P* > 0.95). However, the distribution of members of each of the synthetic diet categories show separation (Fig. 5), and the difference in the average values of the morphological variables of members of each category is statistically significant (*P* < 0.0001). This *P*-value should be interpreted with caution, as the over-representation of the diet category Benthic Arthropods could affect the outcome of the MANOVA (Quinn and Keough 2002). However, the relative phylogenetic rarity and clear separation of each of the remaining diet categories in morphospace supports their biological meaningfulness, which is of course what our study is intending to assess.

While there is variation in the morphospace occupied by members of each diet category, some generalizations can be extracted. Species that primarily prey upon Stationary Benthic Items have short jaws with high anterior mechanical advantage but low posterior mechanical advantage, while species that prey primarily upon Pelagic Arthropods have long jaws. Species that primarily eat Squishy Swimmers have high posterior mechanical advantage, but showed great disparity in anterior mechanical advantage: two of the species (*Hemilepidouts bolini* and *Cottus rhotheus*) have elongate jaws with low anterior mechanical advantage (but high closing velocity, as would be expected for a piscivore), but the third species (*S.marmoratus*) has very abbreviated jaws with high anterior mechanical advantage (Fig. 5). Species that primarily prey upon Benthic Arthropods are confined to an area of morphospace characterized by average PC values and morphologies. As in the phylogenetic distribution of diet types (see Fig. 4), there does not appear to be a strong phylogenetic component to the distribution of species in morphospace (Fig. 5). The possible exception to this observation is again the sister species pair *C.* (*B.*) *globiceps* and *C*. (*B*.) *recalvus*, which have similar morphotypes and diets. The differences in the average values of the morphological variables of the diet guilds when categorized using the coarse diet method are not statistically significant (*P* > 0.29).

We will discuss the remaining results only within the context of the synthetic diet categories, as the differences in trait values among taxa living in freshwater vs. marine habitat or occupying dietary niches defined using the coarse diet method are not statistically significant. The posterior classification of diet categories for each species is ∼85% accurate, with five taxa for whom the posterior classification based on morphology does not match the original classification based on our quantification of the species diet: the freshwater species *Cottus beldingii* and *C. extensus*, and the marine species *Hemilepidotus zapus*, *Porocottus camtschaticus*, and *Rhamphocottus richardsonii*. In the first four cases, the mismatched species were predicted to be classified as preying primarily on Benthic Arthropods, and the fifth case (*R. richardsonii*) was predicted to be classified as preying primarily on Vermes.

## Discussion

### Phylogenetic hypothesis

The topology of our MCC phylogeny is largely congruent with previous phylogenetic hypotheses of our study taxa at the scale of family and subfamily or finer (Kontula et al. 2003; Kinziger et al. 2005; Yokoyama and Goto 2005; Knope 2013; Smith and Busby 2014; Buser and López 2015). This is expected, given that our phylogenetic analysis combined previously published sequence data from many of these studies and analyzed them together. The low posterior probability of many of the basal nodes in our phylogeny is likewise seen in the large-scale analyses of sculpin phylogenetics from which we gathered sequence data (Knope 2013; Smith and Busby 2014). There are a few taxa whose phylogenetic placement herein is unconventional (e.g., *Scorpaenichthys*, *Rhamphocottus*, Oligocottinae), but the low support values in the placement of these taxa in the present and previous molecular studies make their placement herein unremarkable.

The inability to confidently resolve basal nodes within Cottoidea is not unique to the present study but is almost certainly exacerbated by our reliance on strictly mitochondrial genetic loci (see Rubinoff and Holland 2005). However, our results show quite clearly that all the morphological and ecological characters considered herein are highly conserved at the basal nodes and that evolutionary changes are concentrated at the tips of the phylogeny, in areas where our phylogeny closely matches the topology of previous studies. So, while there has generally been disagreement in the precise nature of the basal splits within Cottoidea, this uncertainty is inconsequential in the context of our study.

### Conservative cottids: freshwater sculpins exhibit phylogenetic niche conservatism

Freshwater sculpins demonstrate both phylogeographical and ecological signals of phylogenetic niche conservatism (Wiens and Graham 2005). From a phylogeographical perspective, sculpins have only invaded freshwater twice, once in the Holarctic by the widespread *Cottus* radiation and again in the Nearctic by *M.thompsonii*. Generally, neither of these lineages seem to be experimenting with novel ecological niches as, like most marine cottoids, *Cottus* species feed consistently on benthic arthropods, as does *M. thompsonii* (Wojcik et al. 1986; but see further discussion and the “Atypical cottoids” section). Moreover, *Cottus* are not closely-related to *Myoxocephalus* (Smith and Busby 2014), and therefore fulfill another classic tenet of niche conservatism: that when multiple invasions of a habitat occur within the same clade, invaders are rarely (if ever) closely-related or do not overlap geographically (Yoder et al. 2010; Bloom et al. 2013). Finally, like other marine-derived lineages, once entrenched in freshwater, these sculpins have never left, supporting the idea that while invasions of freshwater are rare, subsequent invasions of freshwater are rarer, and reversals to saltwater are rarest (Vermeij and Dudley 2000; Betancur-R 2010; Bloom and Lovejoy 2012). Niche conservatism appears to be a motif for marine-derived lineages, but while studies have evinced these patterns solely from either geographical or morphological data (Bloom and Lovejoy 2012; rarely both, but see Betancur-R et al. 2012; Davis et al. 2012), our data show geographical, morphological, and ecological concordance regarding constraints on niche lability (Losos 2008) in freshwater cottoid fishes.

Interestingly, most if not all temperate marine-derived taxa have diadromous cousins: clupeiforms, smelt, salmonids, stickleback, and sculpins, appearing particularly well-suited to overcome the geographical and physiological barriers to invading freshwater, while tropical marine-derived lineages are rarely diadromous. However, Bloom and Lovejoy (2014) demonstrate that diadromous fishes which have made the marine–freshwater transition a permanent life history fixture show lower taxonomic diversity than their sister lineages: diadromy as corridor to freshwater environments appears to be an evolutionary dead-end. However, even though freshwater *Cottus* in Europe and Asia (and to a lesser extent in North America) are not morphologically diverse, they are quite speciose (∼100 spp.) and so do not fit the pattern Bloom and Lovejoy (2014) found for clupeiforms. Similarly, more recent work on clupeiforms found similar trophic niches regardless of macrohabitat (marine vs. freshwater) (Bloom et al. 2018). So, what constrains morphological diversification in freshwater sculpins and other similar taxa?

While many marine-derived fishes have limits placed on their diversification by competition with entrenched primary freshwater taxa (Betancur-R 2010; Bloom and Lovejoy 2012), we suggest competition does not stall cottoid diversification in freshwater environments. Why? If diversification in freshwater sculpins is limited by competition with other taxa, then why do they so strongly resemble marine sculpins in terms of bauplan? This would imply that potential competitors are similar in both habitats, which seems unlikely, particularly in depauperate temperate river systems. Moreover, the most diverse freshwater system in which sculpins are found (Baikal), boasts some of the more drastic and novel adaptations of the sculpin bauplan. We find the argument that competition places bounds on diversification in marine-derived taxa to be insufficient in this case, as sculpins overwhelmingly fill the same niche roles in marine and freshwater environments, with some notable exceptions.

Perhaps freshwater sculpins have strong intrinsic (e.g., developmental, phylogenetic) constraints on their niche evolution. The sculpin “lifestyle,” i.e., bound to the benthos without a swimbladder, may constrain the ecomorphology of these fishes in freshwater. Whereas limnetic and epibenthic ecomorphs are common to other marine-derived temperate invaders like stickleback, smelt, and charr (Gislason et al. 1999; Rundle et al. 2000; Barrette et al. 2009) we do not see similar ecomorphs evolving in populations of freshwater sculpins. An intriguing case could be made for an analogous limnetic–epibenthic split, but with the ecomorphs separated by ontogeny rather than population. Many of the species of *Cottus* that occur in lakes (e.g., populations of *C. cognatus*, *C. extensus*, *C. gobio*) have a semi-pelagic juvenile stage, but are epibenthic as adults (Brandt 1986; Ruzycki and Wurtsbaugh 1999; Wanzenböuck et al. 2000). Another analogous example is found in the endemic Baikal cottoids, which show limnetic–epibenthic separation, but across species rather than populations or ontogeny: Baikal oilfishes are limnetic, while their *Cottocomephorus* allies are epibenthic (Sideleva 1996, 2003). Baikal cottoids are an example of sculpins that have adapted very well to pelagic habitats, but we do not see the kind of parallel rapid radiation of morphotypes seen in char and stickleback. However, neither char nor stickleback approach the overall taxon richness of freshwater cottids (∼50/18 vs. ∼100 species, respectively), despite similar geographic ranges (Nelson et al. 2016). Likewise, the lack of a swimbladder in marine sculpin taxa like *Blepsias*, *Vellitor*, *Pallasina*, and *Phallocottus* has not limited their ability to adapt to semi-pelagic habitats. Instead, these taxa differ from the fundamental sculpin bauplan in a major way, as they eschew the overgrown tadpole-like (big head, reduced axial skeleton) sculpin bauplan.

Finally, freshwater sculpins and other temperate marine-derived lineages may be constrained by the very nature of the ecological opportunity they find in novel habitats. Sculpins invade depauperate boreal or temperate faunas, where prey abundances are high, but the diversity of this prey is lower than that of the tropics (Mannion et al. 2014; Heino et al. 2018). Tropical systems like reefs and rainforests have built the most complex ecosystems on Earth from biotic synergy, mutualisms, and specialization, systems whereby diversity begets even greater diversity (Ehrlich 1975; Mouillot et al. 2014). Perhaps the lack of such a biodiversity “critical mass” precludes the ability of freshwater sculpins to become cleaners, lepidophages, or pterygophages, trophic niches that are conspicuously absent from boreal zones or rare in temperate ones (Sazima 1983, 1986). The capacity of ecological opportunity in depauperate systems may be inherently different from tropical analogs, although this certainly has not affected the ability of *Cottus* to spread across wide geographic areas, as they range across Asia, Europe, and North America. Only in diverse systems like Baikal do we see freshwater sculpin depart from ancestral bauplans, an ecoregion known for its ancient and complex diversity.

Alternatively, the close resemblance of freshwater and marine cottoids may not require ecological or developmental constraints but may simply stem from relatively shallow divergence times among these lineages. The simple nature of our phylogeny precludes us from constructing a dated phylogeny, which is necessary for examining rates of diversification in freshwater vs. marine cottoids. However, this does not preclude us from posing hypotheses based on mensurative exercises considering cottoid fossils and dated information from other studies. Initial estimates of the age of the *Cottus* invasion (>1.2 mya in Baikal, >3–4 mya in other *Cottus*) suggest that morphological diversity has had suitable time to accumulate (Kontula et al. 2003; Yokoyama and Goto 2005; Goto et al. 2015). Likewise, the diversification of several freshwater lineages of *Cottus* in North America corresponds with the diversification timeline for percids (Near et al. 2001, 2011), a considerably diverse group ecologically, behaviorally, and morphologically. As such, we propose that freshwater cottoids have had suitable time to diversify and rather that ecological or developmental constraints are more likely to have resulted in modern conservative patterns of their diversification.

### Atypical cottoids

During the transition from marine to freshwater in the *Cottus* lineage, ancestral *Cottus* stock simply shifted from consuming marine Benthic Arthropods to freshwater ones. It would seem then that the ancestral sculpins were preadapted to exploit similar prey resources in freshwater that sculpins were consuming all along in the oceans. The focus on this prey group appears to be not only adaptable to freshwater habitats, but also scalable. In the marine environment especially, small sculpins (e.g., *O.maculosus*, up to 10 cm TL) prey upon small bodied Benthic Arthropods (e.g., gammarid amphipods), medium-sized sculpins (e.g., *Icelinus filamentosus*, up to 27 cm TL) prey on medium-sized Benthic Arthropods (e.g., pandalid shrimp) and large-bodied sculpins (e.g., *Myoxocephalus polyacanthocephalus*, up to 80 cm TL) prey on large-bodied Benthic Arthropods (e.g., brachyuran crabs). While the success of this strategy has apparently resulted in many species of distantly related sculpins that more or less look and act alike despite living in very different habitats, some lineages have nevertheless branched out into novel feeding ecologies and morphotypes. Our results show that taxa which have evolved to eat things other than Benthic Arthropods diverge into unique regions of feeding morphospace and occupy novel habitats. The most extreme example of this is found in the mesopelagic depths of the freshwater Lake Baikal in Siberia. This habitat is home to the Baikal endemic, *Comephorus dybowskii*, the little Baikal oilfish. This fish is an entirely pelagic species and has extremely elongate jaws that aid in sweeping up the pelagic amphipods that make up most of its diet (Miyasaka et al. 2006). The morphological adaptations of *Comephorus* to its pelagic habitat are extreme and constitute a change in bauplan compared with other sculpins (Sideleva 1996, 2003). There are several marine sculpin groups that were not included in this study that, like *Comephorus*, have evolved a much more pelagic lifestyle than the typical cottoid (e.g., *Blepsias*, *Phallocottus*, *Vellitor*) and may likewise prove to be exceptions to the general sculpin bauplan.

Another divergent habitat that contains an unusual sculpin is the high wave-exposure rocky intertidal habitats on the Pacific coast of North America. This habitat hosts *Clinocottus* (*Blennicottus*) *recalvus*, the bald sculpin, and its sister species, *C.* (*B.*) *globiceps*, the mosshead sculpin. The diet of these fishes is dominated by algae (constituting up to 100% of the stomach contents (see Johnston 1954) and their jaws are greatly reduced in length (see Fig. 5 and discussion in Buser et al. 2017, 2018). This jaw shape confers the highest anterior mechanical advantage seen in our dataset, and this is useful for species who use their jaws to grip and then rip macroalgae from its holdfast. This morphology bears a strong superficial resemblance to the intertidal, algae-eating combtooth blennies (e.g., Parableniini), which also variously consume macroalgae (e.g., *Ulva*) and microalgae (e.g., Pennales diatoms) (Hundt and Simons 2018). The extremely rounded profile of the jaws in these particular sculpins and blennies even resembles morphologies seen in freshwater taxa like loricariid catfishes and curimatids (Alexander 1965; Adriaens et al. 2009; Frable 2015), and is advantageous for scraping microalgae off of rocks and other hard surfaces.

In addition to divergence in habitat, many species that are divergent in terms of morphology and diet also differ in feeding behavior. One such taxon is *Hemilepidotus bolini* (bigmouth sculpin), which preys on Squishy Swimmers. This species is a lie-in-wait predator that uses its highly decorated, dorsoventrally flattened body to camouflage itself against the benthos and snap up passing fishes with its long, fast jaws (see Fig. 5). These characteristics, along with its substantial underbite, converge on the morphology and feeding ecology of the anglerfishes in the genus *Lophius*, to which it bears a strong superficial resemblance (Myers 1934).

A final example comes from a species that looks very much like a sculpin, but whose jaw mechanics and feeding ecology are anything but typical: the cabezon (*S.marmoratus*), whose diet is also made up primarily of Squishy Swimmers. This sculpin, however, is much more of a generalist than are the others in its diet guild. Although it preys primarily upon other fishes, *S. marmoratus* also eats a substantial number of crustaceans (e.g., *Cancer* crabs), *Octopus*, and gastropods, including abalone (*Haliotis* spp.) (Burge and Schultz 1973). Given the rather extreme ability of abalone to cling to hard surfaces (i.e., requiring biting and prying ability of would-be predators) it is not surprising that the jaws of *S. marmoratus* have a very high mechanical advantage, both anteriorly and, especially, posteriorly (see Fig. 5). The posterior mechanical advantage is not necessarily indicative of a tendency to crush prey items in the oral jaws, as abalone are swallowed whole and the few sculpin species known to “crush” hard items use their vomer to do so, and actually puncture rather than pulverize (Van Vliet 1964; Norton 1988). Rather, it is likely an indicator of how broadly the oral jaws are used for biting and gripping (see Norton 1991, 1995), perhaps analogous to how clingfishes leverage limpet prey from rocks (Johnson 1970). *S.marmoratus* is the most extreme example of high posterior mechanical advantage, and likely owes this to a need to bite and then grapple with large prey items that necessitate removal from a hard substrate.

### Comparing *Abyssocottus* apples and *Oligocottus* oranges

These observations—that sculpin diet and morphology are generally conserved, but that some lineages have adapted to novel niches and exploited them, are true in both freshwater and marine systems. This overall pattern was not captured by the traditional, coarse system of diet classification, likely owing to its underlying structure being based on the phylogenetic relationships of the prey items, rather than their traits. While insects, for example, are primarily restricted to freshwater systems, the aquatic larvae of, e.g., mayflies (Ephemeroptera), function very much like many herbivorous amphipods and marine isopods, and these similarities may have in fact been a contributing factor to the sculpins’ successful invasion of freshwater systems, essentially following a niche that transcends the marine–freshwater interface. Our method of prey categorization provides a completely explicit, data driven approach to diet classification that not only represents the actual diet of species accurately, but is capable of translating a large pool of prey items into a manageable number of categories and thus facilitating comparisons of otherwise highly divergent feeding ecologies across a common metric. As in any data-driven approach, however, this method is dependent on the quality of the underlying data, especially on the original diet descriptions for each species.

The posterior classification of diet categories provides a test of the initial synthetic diet categorization. There were only five mismatches between these systems, but these exceptions offer insight into some interesting aspects of biology and ecology that are difficult to capture in any generalized model of functional feeding morphology. In the case of the mismatched species, *C.extensus*, the misclassification could be the result of a known shift in habitat between adults and juveniles. Adults are benthic, but juveniles are pelagic (Ruzycki and Wurtsbaugh 1999). It could be that the adults switch to benthic prey when they shift habitats, but the diet of adults is not known. Alternatively, the morphology of *C. extensus* may simply be an ancestral condition that has yet to “catch up,” so to speak, with the presumably novel niche of planktonic prey found in Bear Lake, Utah, to which *C. extensus* is endemic. Virtually all of *C. extensus*’ closest relatives feed primarily on Benthic Arthropods. The other cases are more nebulous but may likewise reflect a gap in our understanding of the diet of these species, or perhaps be indicative of outstanding behaviorally- or physiologically-mediated prey use (i.e., in lepidophagous fishes: Hahn et al. 2000; Janovetz 2005; Kolmann et al. 2018) or predators feeding across multiple diet categories (Day et al. 2011; Lujan et al. 2011).

## Conclusion

Regardless of whether they live in marine or freshwater habitats, many sculpins look remarkably similar and perform similar ecological roles. This mirrors the sort of phylogenetic niche conservatism that others have found in marine-derived freshwater lineages in the Neotropics; however, we document these patterns in a Holarctic clade of fishes and explicitly tie feeding morphology to dietary ecology. Whereas most sculpins are adapted for consuming Benthic Arthropods (regardless of habitat), notable exceptions include taxa like the freshwater Baikal oilfish (*Comephorus*, a pelagic planktivore), marine *Hemilepidotus* (a sit-and-wait piscivore), and *Clinocottus recalvus* (an intertidal herbivorous grazer). These taxa represent astounding trophic novelties in a clade of largely benthic invertebrate feeders, highlighting that transitions between habitats (at least for Baikal oilfish) may not change the overall diversity of marine-derived lineages, but can produce isolated ecological novelty. We also classify diet categories using a novel, quantitative approach based on clade-specific data, rather than traditional qualitative prey categories. This method resulted in better fit between our morphological data and dietary categories over more traditional categories. We propose that this method reduces bias by eliminating a tendency in the literature to both wedge species into ill-fitting ecological boxes or separate similar functional categories of prey (e.g., fish and squid) based on taxonomic, rather than practical, considerations. Additionally, this method categorizes prey using functional traits, giving us some deeper perspective into predator prey-interactions, from morphological and behavioral standpoints.

## Supplementary Material

obz023_Supplementary_Data
