# Supplementary figures and images for: Have Niche, Will Travel. New Means of Linking Diet and Ecomorphology Reveals Niche Conservatism in Freshwater Cottoid Fishes
Source: Integr Org Biol. 2019 Sep 6;1(1):obz023. doi: 10.1093/iob/obz023 (PMC7671106; doi:10.1093/iob/obz023)

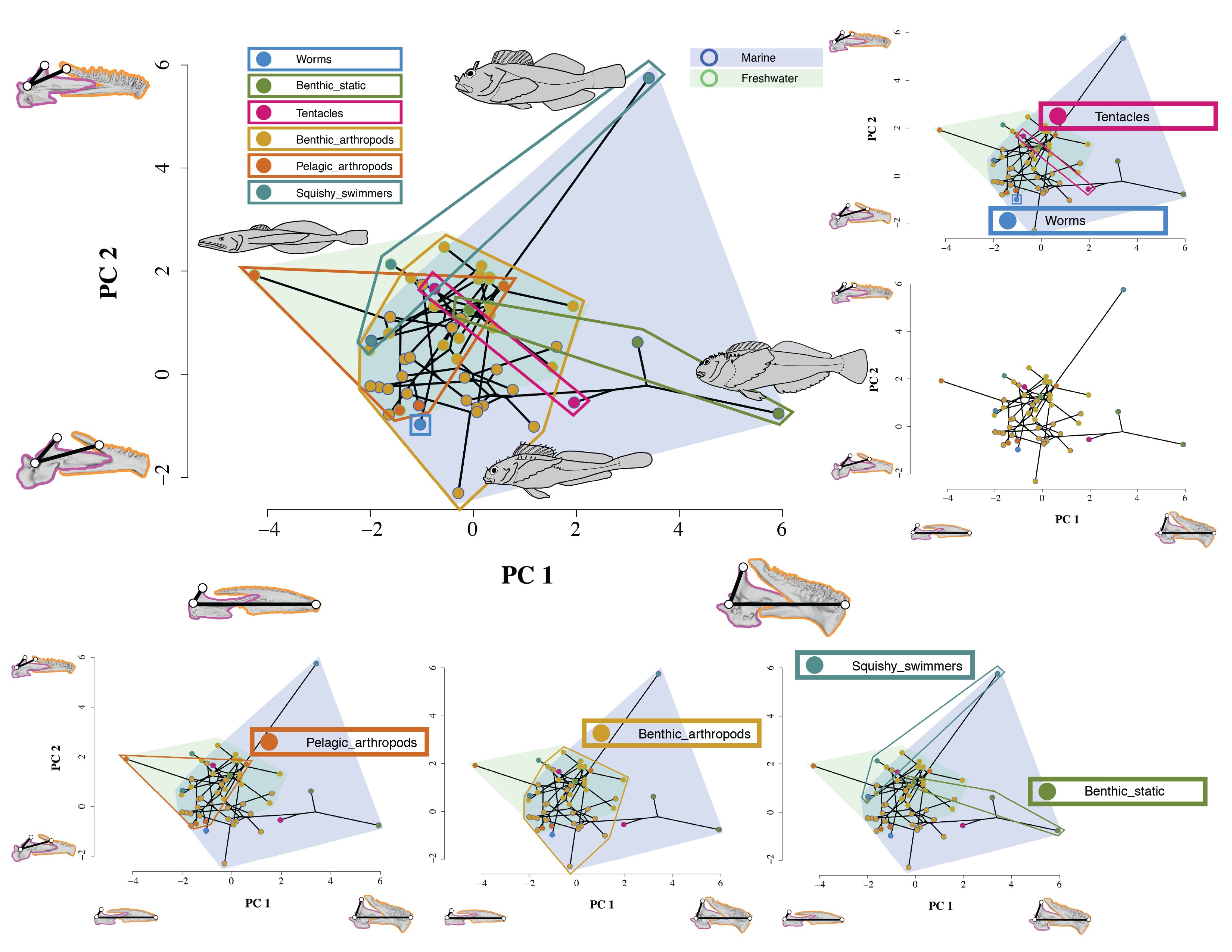

Supplement: obz023_Supplementary_Data [file obz023_supplementary_data.zip › Supplementary Data 4_Phylomorphospace_panels.png]
